# Supplementary figures and images for: Autozygome Sequencing Expands the Horizon of Human Knockout Research and Provides Novel Insights into Human Phenotypic Variation
Source: PLoS Genet. 2013 Dec 19;9(12):e1004030. doi: 10.1371/journal.pgen.1004030 (PMC3868571; doi:10.1371/journal.pgen.1004030)

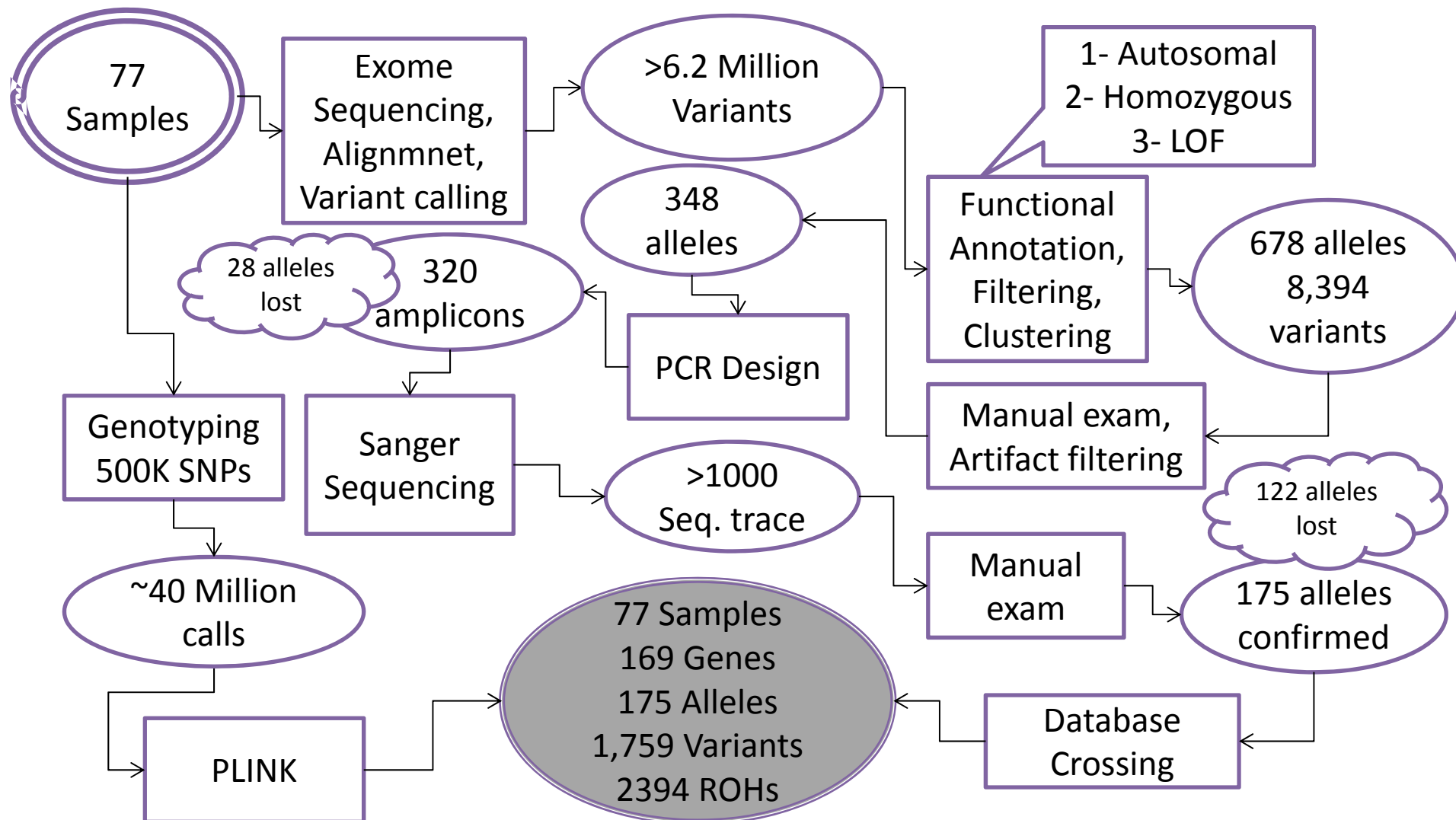

Supplement: Figure S1 — The data flow for extracting homozygous LoF alleles from exome NGS data. (PDF) [file pgen.1004030.s001.pdf]
