# Supplementary material for: Autozygome Sequencing Expands the Horizon of Human Knockout Research and Provides Novel Insights into Human Phenotypic Variation
Source: PLoS Genet. 2013 Dec 19;9(12):e1004030. doi: 10.1371/journal.pgen.1004030 (PMC3868571; doi:10.1371/journal.pgen.1004030)
Supplement: Text S1 — Supplementary methods (extracting ROH blocks, NGS data pre-processing, candidate allele verification and scoring, variant validation by sequencing, heterozygous variant validation, database crossing and data integration, linking to HGNC and OMIM, linking to EVS, frequency distributions for different variant classes, determining LOF discovery efficiency as a function of ROH minimum length, and allele frequency vs. autozygosity). (DOCX) [file pgen.1004030.s002.docx]

Supplementary Material for

**Autozygome Sequencing Expands the Horizon of Human Knockout Research and Provides Novel Insights into Human Phenotypic Variation**

**Supplementary Methods:**

Extracting ROH blocks:

Genomic DNA was extracted from blood samples for each of the 77 individuals and genotyped on Axiom Genome-Wide Human SNP microarrays, following standard protocols. Microarray data analysis and genotype calling was performed using manufacturer’s software on default settings. SNPs where mapped to the hg19 genome release using the “Axiom_GW_Hu_SNP.na32.annot.db” annotation database. In comparing that annotation DB to an earlier version (Axiom_GW_Hu_SNP.r2.na29.annot.db, mapped to hg18) we detected a small number (114) of probes that seemed to have been mapped to different chromosomes between the two DBs and so we flagged them as potentially unreliable probes. All data was then combined and properly formatted for input into PLINK (<http://pngu.mgh.harvard.edu/~purcell/plink/>) using a simple PERL script. At this stage we excluded probes mapping to chromosomes X and Y and the earlier flagged probes. For efficient processing, PLINK was used to reformat the data into a compact binary format and to also filter out probes that failed (a NoCall) in >10% of the samples (about 5000 probes), using the “--geno 0.1 --make-bed” flags. After all filtering, we had 543,995 SNPs per file and the genotyping rate over all individuals was 97.9%.

We then ran PLINK in ROH detection mode with the following parameters:

--homozyg-gap 1000

--homozyg-density 50

--homozyg-snp 20

--homozyg-kb 100

--homozyg-window-kb 5000

--homozyg-window-snp 20

--homozyg-window-het 3

--homozyg-window-missing 1

--homozyg-window-threshold 0.05

The parameter set essentially calls all ROH blocks 100KB or longer with a minimum average homozygosity rate of 85% and no gaps longer then 1MB, at sensitivity levels compatible with our chips. ROHs were called at an unusually low cutoff to allow efficient selective filtering at later stages of the analysis and to test the relationship between ROH length and LoF detection efficiency. The resulting data, essentially a list of chromosomal blocks for each individual, was loaded into our unified database model for further processing.

NGS data preprocessing:

Each of the 77 exomes was aligned to the hg19 genome release using BWA (<http://bio-bwa.sourceforge.net/>), followed by variant detection and calling using SAMTOOLS (<http://samtools.sourceforge.net/>). Further processing to add functional annotation and dbSNP links was carried out using ANNOVAR (<http://www.openbioinformatics.org/annovar/>) utilizing the RefSeq gene models. These steps were performed by a number of collaborators at different times resulting in a somewhat mixed collection of software versions and annotation DB releases.

Each annotated exome was supplied in a separate MS Excel file with a somewhat differing feature set of columns. The data was therefore loaded onto a unified central MS Access database after manual inspection of each file and correction of some artifacts. The use of a mixed collection of software versions and annotation DB releases resulted in various inconsistencies in gene naming, variant calling and localization and impact prediction that resulted in major difficulties with later analysis, particularly while clustering reported variants into a representative allele. An interesting example occurred due to the use of 0-based and 1-based coordinate systems in different files. Countermeasures to manage this technical issue resulted in hiding an underlying biological issue whereby in some cases, two adjacent SNPs in the same codon are always coupled (become an MNP) and their combined impact on the ORF is usually different from what is predicted if each SNP was evaluated independently. Even though we were aware of the MNP issue, the preprocessing step hid most of its instances and this was not detected and corrected until later stages when it was realized that MNP artifacts remained in our data even though we attempted to filter them out.

To overcome gene naming and impact prediction vocabulary inconsistencies, additional auxiliary tables were used to map the various forms into unified entries. Inconsistencies in localizing called indels were handled manually in later stages to combine very similar alleles after manual inspection.

Candidate allele verification and scoring:

After the initial data consolidation and cleanup, the DB was queried to produce a list of potential LoF alleles and their frequencies. This was now a simple selection filter for autosomal (not in chr. X or Y), homozygous, LoFs (stop-gain or frameshift in a protein coding ORF); followed by grouping on variant position and type. The result is a set of 678 candidate alleles from 8394 reported variants (across all 77 exomes). A simple pipeline to facilitate manual inspection was then setup to bring up each of these alleles on the UCSC genome browser (<http://genome.ucsc.edu>/) in a suitable zoomed-in context and annotation tracks, and to allow storing controlled notes about these alleles. This manual step was necessary due to the complex nature of the human transcripts, the need to integrate multiple sources of evidence nonlinearly, the presence of annotation errors both in the reference gene models and in the reference human genome, and the presence of some inconsistencies in the NGS annotation pipeline.

The aims of the manual inspection were to:

1. Verify each reported LoF allele by examining both the genomic context and conservation (to detect possible reference genome sequence errors), and the transcriptomic context to detect the presence of alternative splicing and its interaction with the allele. We also examined apparent high variation around the examined allele and excluded cases where the gene appeared highly variable.
2. Detect potential rescue mechanisms for each candidate LoF allele, such as alternative splice sites, nearby repeated fragments, or potential different ORF start sites. Also, each allele was examined against a full variant report listing for each candidate all variants (including non-LoF and heterozygous variants) nearby, grouped by each individual. This allowed for detecting alleles that might interact in the same individual to mitigate the expected effect of the reported LoF variant (for example: two adjacent and coupled SNPs (i.e., MNP), a frame-shifting insertion followed by a frame-restoring deletion or two consecutive deletions that together become non-frame-shifting, and so on).
3. Summarize the impact-assessment of the allele using a simple integer score that expresses the potential for the LoF variant to ablate the gene product and therefore provide a means for ranking the various LoF variants at later stages. A score of 3 was given to alleles that would affect all gene transcripts and where the LoF would impact more than 10% of the ORF. A score of 2 was given to alleles that would impact more than 10% of the ORF but only affected some of the gene’s transcripts, or alternatively, an LoF that affected all transcripts but only towards the end of the ORF. The lowest score of 1 was given to LoF variants that would only affect the last 10% of the ORF and only in a subset of the gene’s transcripts. A score of 0 indicates that the candidate should be disqualified and excluded from further analysis.

Variant Validation by sequencing:

Following the verification step, 348 alleles were scored above 0 and we proceeded to confirm them by Sanger sequencing. The alleles were examined in larger genomic context to determine a suitable sequencing target and primers were designed using the Primer3 (<http://primer3.wi.mit.edu/>) web tool. In 28 cases no suitable primers could be designed or the primer pairs resulted in non-specific amplification and so the alleles were also excluded from further analysis. PCR amplification was used to extract variant amplicons from 3 randomly selected individuals among those in which the variant was reported. If the variant was reported in 3 or less individuals, all reported cases were PCRed. All amplicons were then submitted for Sanger sequencing. In some cases the PCR or sequencing reactions did not work properly and a repeat or new sample was prepared. In one case, we sequenced amplicons from all 77 individuals to confirm a 100% coupling of one deletion and one complementary insertion in the SON gene that combined resulted in a 4 amino acid substitution mutation instead of the expected frameshift effect expected from each individually. SON was consequently disqualified from our report.

Overall we performed over 1000 sequencing reactions and examined each resulting trace file manually to confirm the presence of the reported homozygous variant. If the variant could not be confirmed in only one of the examined individuals, a substitute was sequenced. If two or more sequencing results failed to confirm the variant the allele was excluded from further analysis. Of course, variants reported in only one individual were either accepted or rejected based on one sample.

About 40% of the alleles were excluded and 195 alleles were confirmed. The experimental results were performed by a number of people and reported in various MS Excel files and needed to be manually examined and reconciled. The results were loaded into the database for crosslinking and further analysis.

Heterozygous variant validation:

Heterozygous variants were selected from the databases and grouped as above with the homozygous variants. Two additional filters were used. One based on the NGS reported quality score (Q>70; based on personal experience with the platform) was imposed to reduce false positive reports. The other was based on association with an autosomal recessive disease, according to OMIM (explained later).

The alleles were not manually examined or scored and were directly validated by Sanger sequencing. We detected candidate alleles in 327 genes. After attempting >70% of the alleles and manually examining more than 300 sequence trace files, we only confirmed a subset in 43 genes. Many of the disqualified variants were only reported in one or two cases and appeared novel, with no prior reports in dbSNP.

Database crossing and data integration:

Results from the manual scoring and sequencing based confirmation were combined with the initial candidate variants lists (homozygous and heterozygous) to produce a confirmed variants lists (CVL). The homozygous CVL contains 175 alleles in 1759 variants. The heterozygous CVL contains 52 alleles in 144 variants. The key idea in the CVL is that for a variant reported in more than 3 individuals and confirmed in a subsample of at least 3, we consider the NGS report fully accurate and thus consider the rest of the variants (reported but not tested) as confirmed. In addition, we combined 20 of the 195 confirmed homozygous LoF alleles with other variants in the list after manual inspection and determining that the different variants were very similar in nature and effect (eg: combined a T/C and a single T/G report into one allele). This helped give a more accurate view on the prevalence of some LoF variants compared to the more fragmented set. The combined variants were denoted in the reported list.

The CVLs were next crosslinked to other resources such as OMIM, the ROH list, and the EVS db to produce the reported tables, figures and summary statistics. Allele ancestral state was extracted from the UCSC Multiz alignment track with the Chimp genome for SNP alleles only (no ancestral state was evaluated for indel alleles).

Linking to HGNC and OMIM:

Gene name mapping data was downloaded from HGNC (<http://www.genenames.org/>; full set) on the 30^th^ of July, 2012. The data was loaded into our database and mainly used for gene name mapping of updated symbols.

Annotations from OMIM (<http://www.omim.org/>) were fully downloaded to a local server on the 9^th^ of July, 2012. Simple PERL scripts were used to preprocess the files and extract relevant information for loading into our database. Mapping of gene identifiers to OMIM ID’s was mostly automatic, using OMIM mapping files. In some cases, due to gene naming updates, automatic mapping was not possible and these cases were augmented manually.

Because OMIM has now expanded beyond mapping genes to Mendelian disease phenotypes, we further processed OMIM phenotype annotations to distinguish susceptibility and relatively benign phenotypes from more severe clinically notable disease phenotypes. We used simple keyword analysis for the terms “deficiency”, “familial”, “syndrome”, “congenital”, “malformation”, “susceptibility to” and “resistance to”. This was augmented by manual classification for about 50 phenotypes.

Genes linked to a disease phenotype (as determined above) were used to build the heterozygous CVL and to detect unexpected results in the homozygous CVL which were reported and discussed in the main text.

Linking to EVS:

The Exome Sequencing Project dataset ESP6500SI was downloaded from the Exome Variant Server (<http://evs.gs.washington.edu/EVS/>) to a local server and processed with a simple PERL script in preparation for loading into our database. Essentially we extracted a slightly summarized view of the dataset removing non-autosomal alleles and redundant reports (by grouping on allele position and mutation type). We also anchored the variant calls to the reference genome allele and calculated the observed genotype frequencies (i.e, the frequency of the ref. allele, the alternative allele and the heterozygous allele).

The EVS was then linked to our homozygous CVL based on positional matching (to within a ±3 basepairs tolerance) and variant type. This was necessary so as not to lose EVS reports for variants called slightly differently from our variants and shifted in position. Alleles in the CVL that matched more than one EVS allele were manually examined to select and keep the correct match.

Frequency distributions for different variant classes:

We first selected all autosomal homozygous variants reported by the NGS and grouped them by position and type to obtain frequency data for all alleles. Allele frequencies over 50% were complemented (100% - freq) to measure the minor allele frequency and avoid spikes caused by annotation errors. Alleles were then grouped by gene and class (synonymous, non-synonymous, non-sense, frameshift, or intronic) keeping the maximum frequency, within each gene, for each class (for example, if gene XYZ contained 10 intronic variants and 3 synonymous substitutions, we keep only the most frequently reported intronic variant and the most frequently reported synonymous variant). This step normalized the data against bias from gene size and/or gene-level selective pressure. The collection of frequency measures for each class was then exported to a statistics software package (Prism v5.03; <http://www.graphpad.com/>) for statistical analysis and plotting.

We also carried out the same analysis on EVS data (data not shown) and observed closely similar patterns and significance levels, except that all frequencies were much lower. The reason for the shift in frequencies is the small sample size for our data compared to the EVS sample (77 vs. about 6500 respectively) and the integer effect on small samples (the data are basically counts). To confirm this explanation, we re-scaled the EVS data to a sample size of 77 individuals, rounding fractions to integers, and then reapplied the analysis. The results matched our data very closely.

Determining LoF discovery efficiency as a function of ROH minimum length:

Because ROH data was extracted at a minimum length cutoff of 0.1 MB from the genotyping data, we were able assess the effect of varying the minimum cutoff efficiently within our database without repeatedly running PLINK to extract the ROHs.

At a minimum cutoff of 0 (no ROH effect), 100% of the autosomal genome is captured and 100% of all reported LoF variants (1759) are included. The resulting gain is 100/100 - 1 = 0%.

At say the 4MB cutoff (i.e, only ROHs of 4MB or longer are included) only about 6% of the genome is covered, but about 10% of all LoF variants that are there to be found are captured, and therefore the gain is about 75% above random (one would have expected to recover only 6% of the LoF variants, instead of 10%).

As the minimum cutoff is raised, fewer ROH blocks are included, though of a larger size. The drop in the number of blocks is exponential, compared to a linear increase in the block size and the overall effect is a near exponential drop in the proportion of the genome included within ROHs of the indicated minimum length. The graph reports the average genome coverage across the 77 individual genomes analyzed.

If the LoF variants were distributed randomly across the genome, the fraction of variants observed within an ROH of the indicated minimum cutoff should almost equal the fraction of the genome covered. This however is not the case and the fraction of LoF variants covered is almost twice the fraction of the genome covered. This indicates that LoF variants are not randomly distributed across the genome, and the graph shows that no matter how one decides to select the cutoff there will be a smaller fraction of LoF variants lost than the fraction of genome excluded. In large population screening efforts, focusing on the autozygome thus translates to major efficiency gains. A double advantage results from the observation that the rarer variants, which would require screening larger samples, are more likely to fall within an autozygous block than outside of it.

Allele frequency vs. autozygosity:

Even though we determined that LoF variants are more likely to be found within the autozygome, the profile of the variants was not clear. We hypothesized that rare alleles are more likely to be private and thus fall within the autozygome than outside of it. To test this, we first called the autozygome at an ROH minimum length cutoff of 2MB for each of the 77 individuals. We next looked at each of the alleles in our CVL and determined the number of times it was observed within the autozygome of the affected individual or outside of it. We assume that this split is a random process that is binomially distributed. From the mapping efficiency analyses, we already knew that 14.4% of all detectable LoF variants were within an ROH of 2MB or longer and used this frequency as the prior for the random binomial process. We did not use the genomic coverage fraction (7.4%) as the prior because we already knew a certain gain over random split (95%) existed and wanted to detect bias effects above and beyond that gain.

Using the binomial random process with a prior of 0.1444, we estimated the probability of observing the in/out split for each variant by random chance and focused on those variants that appeared to be significantly biased towards being either inside the autozygome or being outside of it, at p<0.1. To minimize the integer effect on small sample sizes, we excluded variants observed at less than 3/77 times overall. We then collected the overall (unsplit) frequencies for variants found to be significantly biased to be outside the autozygome (n=12) and those biased to be inside (n=11). The two frequency distributions were compared graphically on a box plot and using the non-parametric Mann-Whitney rank test for median differences, which were found to be significantly different at p<0.0005. The test was repeated (data not shown) at different ROH minimal length cutoffs with similar results in all cases.
